# How to Prioritize Energy Efficiency Intervention in Municipal Public Buildings to Decrease CO_2_ Emissions? A Case Study from Italy

**DOI:** 10.3390/ijerph17124434

**Published:** 2020-06-20

**Authors:** Filomena Pietrapertosa, Marco Tancredi, Michele Giordano, Carmelina Cosmi, Monica Salvia

**Affiliations:** 1 Institute of Methodologies for Environmental Analysis-National Research Council of Italy (CNR-IMAA) C.da S. Loja, 85050 Tito Scalo (PZ), Italy; carmelina.cosmi@imaa.cnr.it (C.C.); monica.salvia@imaa.cnr.it (M.S.); 2 Lucanian Energy Company-SEL. Corso Umberto I, 28, 85100 Potenza PZ, Italy; marcotanc@gmail.com (M.T.); michele.giordano@selspa.it (M.G.)

**Keywords:** public buildings renovation, public buildings energy optimization, energy efficiency, decision-making support tool, Mediterranean (MED) regions

## Abstract

The European Union 2050 climate neutrality goal and the climate crisis require coordinated efforts to reduce energy consumption in all sectors, and mainly in buildings greatly affected by the increasing temperature, with relevant CO_2_ emissions due to inefficient end-use technologies. Moreover, the old building stock of most countries requires suited policies to support renovation programs aimed at improving energy performances and optimize energy uses. A toolbox was developed in the framework of the PrioritEE project to provide policy makers and technicians with a wide set of tools to support energy efficiency in Municipal Public Buildings. The toolbox, available for free, was tested in the partners’ communities, proving its effectiveness. The paper illustrates its application to the Potenza Municipality case study in which the online calculator DSTool (the core instrument of the toolbox) was utilized to select and prioritize the energy efficiency interventions in public buildings implementable in a three-year action plan in terms of costs, energy savings, CO_2_ emissions’ reduction and return on investments. The results highlight that improvements in the building envelopes (walls and roofs), heating and lighting and photovoltaic systems allow reducing CO_2_ emission approximately 644 t/year and saving about 2050 MWh/year with a total three-year investment of 1,728,823 EUR.

## 1. Introduction

The European Union (EU) as a signatory of the Paris Agreement [1], aims to reduce greenhouse gas emissions by at least 40% by 2030 compared to 1990 [2]. The achievement of this ambitious target is supported and reinforced by the European Green Deal, published by the European Commission on 11 December 2019, whose overarching objective is to reach net-zero greenhouse gas emissions by 2050 in order to make the EU the first climate-neutral transnational region.

Cities, representing more than 70% of global CO_2_ emissions [3] and hosting 55% of the world’s population [4], play a key role in driving the transition towards a low carbon society [5] and are called on to scale-up their efforts [6] by defining and implementing sound mitigation and adaptation actions to support greenhouse gas (GHG) emissions reduction.

The building sector is critical to meet the demanding climate neutral target, as it accounts for about 40% of final energy consumption and 36% of CO_2_ emissions in Europe, heating and cooling accounting for about 50% of the annual energy consumption [7].

Moreover, it is estimated that temperature variations, rapid urban development and higher living standards [8] will cause an increase in the energy demand of buildings with a shift between dwelling heating and air cooling share, in particular in hot and humid countries [9], affecting the security of energy supply and the stability of the electrical systems [10].

This means that local policies should target buildings as a key sector, reducing their energy consumption and improving their performances to achieve a drastic reduction in CO_2_ emissions (e.g., [6,11]). Considering that around 75% of the EU building stock is energy-inefficient [7], there is a high untapped energy saving potential that can be activated through technical interventions and energy efficiency measures. Energy renovation of buildings represents, therefore, a key strategy as also highlighted in the key actions of the European Green Deal roadmap for a “Clean, affordable and secure energy” [6] that calls for a “renovation wave” of public and private buildings to make them more sustainable and resistant to the effects of climate change. 

This is in line with the new long-term renovation strategies [12] of Europe aimed at decarbonising the national building stocks by 2050. The framework put in place by Europe to speed up the renovation of buildings were set up in the Directives on Energy Efficiency (EED, 2012/27/EU [13]), and on Energy Performance of Buildings (EPBD, 2010/31/EU [14]), and in the subsequent amendments ((EU) 2018/2012 [15]) and (2018/844/EU [11]) that are part of the Clean Energy for All Europeans package. 

The directives are strongly interconnected, and both are aimed towards boosting the energy performance of buildings by promoting policies aimed to achieve a highly energy-efficient and decarbonised building stock contributing to reach carbon neutrality by 2050.

On the way to 2050, each Member State has to define sound strategies in its National Energy and Climate Plan (NECP), setting energy efficiency goals for 2030, 2040 and 2050, in particular for the building sector.

Among the measures aimed at improving the building stock, the new EPBD [11] requires that all new buildings must be nearly zero-energy buildings (NZEB) from 31 December 2020 (for all new public buildings, this obligation is brought forward to 31 December 2018). Moreover, it sets the cost-optimal minimum energy performance requirements for existing buildings that undergo major renovation and for the replacement or retrofit of building elements like heating and cooling systems, roofs and walls, as it requires EU countries to draw up a list of the national financial measures that can support an improvement in the energy efficiency of buildings.

In this context, renovation of Municipal Public Buildings (MPBs) is particularly important to reduce both CO_2_ emissions and the energy bill of a local council. In MPBs, energy-saving potential can be exploited by promoting suitable energy requalification measures and the use of renewable energy technologies. However, the identification of cost-effective energy measures should be done considering not only the energy-saving potential and the expected CO_2_ reduction, but also the economic feasibility in terms of necessary total budget and return on investment (ROI). A key issue is, therefore, to make available and provide public authorities with tools that can be used to support the decision-making process by allowing a preliminary classification of interventions based on multiple quantitative indicators.

Such tools should be user-friendly, open-source and easily transferable to energy planners, policymakers, and local administrators, after a short “hands-on” training, to make them actually used in the activities of policy design, implementation, and assessment.

There are a significant number of tools and information sources tackling energy-related topics, such as energy planning and energy efficiency [16]. It is therefore necessary to provide policy makers with a suited easy-to-use tool which takes into account the policy objectives, the intended use, the investigated sectors (sub-sectors), data availability, user friendliness, skills and motivation of the staff personnel involved, in order to minimise the required efforts for data gathering and analysis, valorising synergies and promoting participation and an effective organisational learning.

The application here presented is considered particularly pertinent in the current scenario, in which the international framework is increasingly recognizing and fostering the role that MPBs must play in reducing energy consumption. In the framework of the Interreg MED PrioritEE project “Prioritise energy efficiency (EE) measures in public buildings: a decision support tool for regional and local public authorities” [17], an easy-to-use Decision Support Tool was developed, which is one of the main components of the PrioritEE Toolbox and was applied to determine the priority energy efficiency interventions in public buildings in five case studies in the Mediterranean region: Karlovac County (Croatia), Municipality of Potenza (Italy), Aragón region (Spain), Lezíria do Tejo Intermunicipal Community (Portugal), and Region of Western Macedonia (Greece). This paper focuses on the application of the DSTool to the case study of the municipality of Potenza (Southern Italy), where selected municipal public buildings were characterised and analyzed to identify the priority interventions to improve energy efficiency and optimize energy uses, evaluating their effectiveness in term of energy savings, reduction in CO_2_ emissions and return on investment (ROI). The ultimate goal was to define a local action plan that could support the drafting of the city’s Sustainable Energy and Climate Action Plan (SECAP) that the city administration would like to implement starting from the previous Sustainable Energy and Action Plan (SEAP) [18]. 

## 2. Data and Methods 

Based on an exhaustive review of available tools carried out in [16] and in the framework of the PrioritEE project [17], a toolbox including a decision support tool was implemented ad-hoc during the project with the aim to support local authorities and decision makers in the selection and prioritization of investments to increase energy efficiency and the use of renewable energy technologies in Municipal Public Building. 

### 2.1. The PrioritEE Toolbox

**Figure 1:**
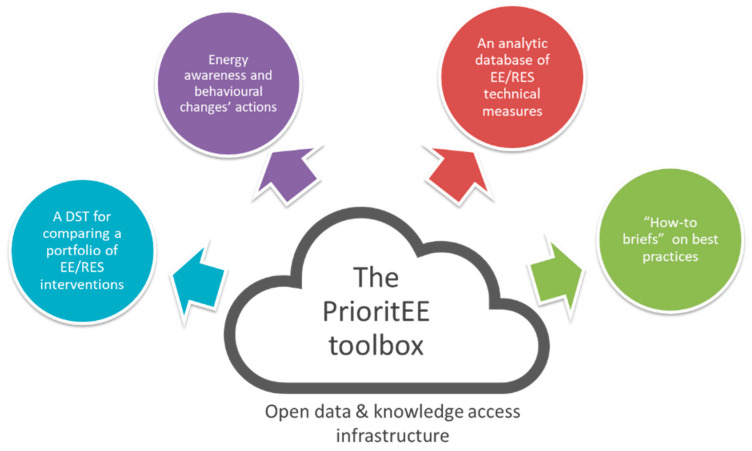
Main components of the PrioritEE Toolbox.

The Decision Support Tool (DSTool) is one of the core components of the PrioritEE toolbox (Figure 1), which was designed with the aim to improve the capacity building of the Public Authorities in the energy management of MPBs. The other components of the toolbox are: an Analytical Database of energy technologies; a Repository of Good Practices on energy efficiency technical and behavioral related topics; seven How-To-Briefs with brief technical and practical information; an open data and knowledge infrastructure which links all the four components, making them freely available through the PrioritEE project website [19]. The development of each component of the Toolbox was coordinated by one of the scientific partners involved in the PrioritEE project. In particular, the DSTool was mainly designed by REGEA, the North-West Croatia Regional Energy Agency, and all the national teams (including the Authors as concerns the Italian case study) provided feedback, national average data and technologies, tested it and helped to refine it according to the feedback received by local users [17].

An introduction to the toolbox components can be found in [20,21]. In particular, they aim at providing a concrete help to local authorities and decision makers in all the phases involved from the identification and prioritization of the most appropriate energy-efficient interventions till the definition of energy–environmental policies and action plans on different timeframes.

In the initial phase of energy planning, the how-to-briefs can provide some basic information on several themes of interest: energy efficiency interventions in buildings, how to design a plan, select and involve stakeholders, interact with the target groups and which programmes can support the financing of actions. More precise information on the technical options can be found in the analytical database on Energy Efficiency (EE) interventions and Renewable Energy (RES) technologies. In addition to that, concrete ideas on how building users can increase energy saving in different types of Municipal Public Buildings can be inspired by the repository of good practices for changing energy behaviour collected across Europe [22]. 

After examining the preliminary context, the Decision Support Tool (DSTool) allows for characterizing the overall set of MPBs, selecting the ones to be renovated, identifying the appropriate technical interventions and evaluating the potential investment opportunities in terms of different parameters, as described in detail in [20], and briefly recalled here.

*The PrioritEE* DSTool was firstly developed as a ***spreadsheet-based tool*** structured around four main components (Basic Inputs, Advanced Inputs, Calculations, and Results) It was designed by REGEA to support local authorities in developing sustainable energy action plans. It was conceived in order to be adapted to different user needs, climate, energy use profile, building characteristics and regulation.

A two-level approach (Basic Data and Advanced Data) characterises the data input, allowing a customised utilization of the tool, i.e., based on the user’s technical skills and availability of data on the buildings under focus, e.g., as concerns energy consumption per final energy carrier and end-uses. The two-level input structure allows users to analyze their buildings and get more accurate results when detailed input data are provided. At the same time, a basic database with country- and regional-specific parameters (average temperatures, prices, taxes) is available for each of the five PrioritEE partner countries (Croatia, Greece, Italy, Portugal and Spain) to compensate for missing data where necessary. Six types of buildings are considered (offices, educational buildings, cultural buildings, social centres, sports facilities, and swimming pools). The Calculations section estimates the current building status according to several indicators necessary for benchmarking the EE and RES interventions for each municipal public building and for the whole stock of buildings considered. Energy efficiency measures are divided among nine intervention areas: thermal insulation of the external walls (coat); replacement of fixtures (windows); thermal insulation of the roof and ceilings; ventilation system; heating system; air cooling system; lighting; thermal solar; photovoltaic (PV) system.

The DSTool produces a ranking and prioritization of EE and RES interventions in each of the selected MPB as the results; per-measure or per-combination of all possible EE and RES interventions in the nine areas considered. The ranking of priority of buildings to be targeted is made according to heat and electricity savings while, for each of the selected buildings, four key performance indicators (energy saving (kWh), avoided CO_2_ emissions (t/year), investments (EUR), return on investment (ROI) (years)) are calculated. These results allow for classifying and establishing the priorities of the EE and RES interventions in each building and in the whole stock of MPB, considered on the basis of different criteria and time horizons. More details on the technical features of the DSTool can be found in [23]. 

**Figure 2:**
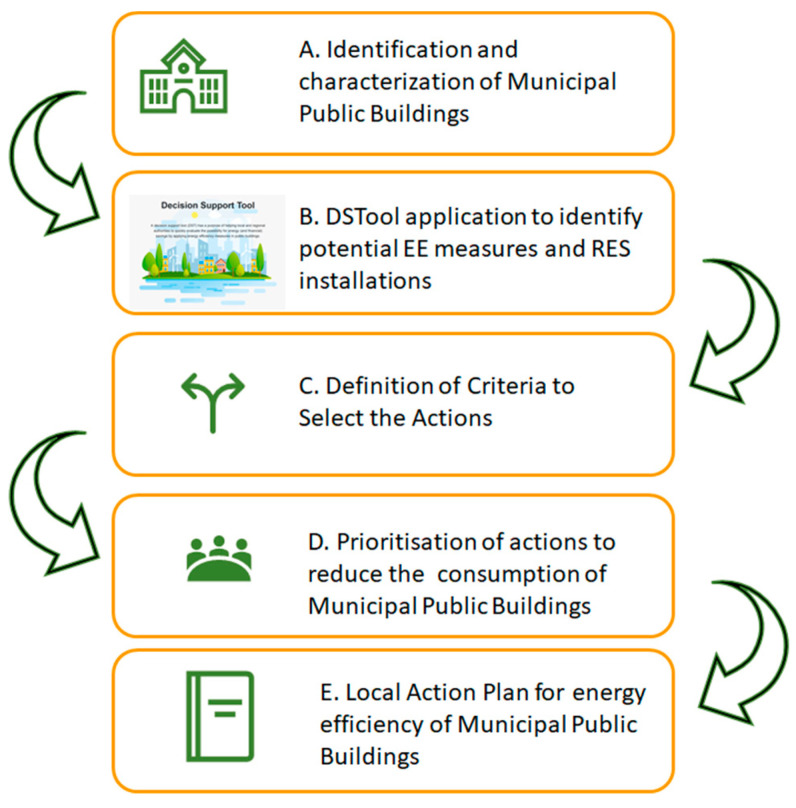
Step-by-step procedure to implement a Local Action Plan using the PrioritEE DSTool.

The initial spreadsheet version of the PrioritEE DSTool was then converted into an interactive web-based technical calculator, available through the project’s website [24] and can be freely used as a key element for obtaining quantitative indications for the selection of technical measures to reduce CO_2_ emissions, which constitutes the basis to draw up a Local Action Plan (Figure 2) [25].

### 2.2. The Case Study of Potenza Municipality 

This procedure was tested in the Case Study of Potenza Municipality, a city in Southern Italy with a population of about 67,000 people (2016 census). The municipality, located 800 m above sea level on the Apennine Mountains of Lucania, is the highest Italian regional capital. A mountain Mediterranean climate, warm and dry in summer and cold and snowy in winter, characterizes the territory. 

Potenza belongs to the climatic zone “E”, one of the colder climatic zones calculated in compliance with the Italian regulation on heating systems in buildings (D.P.R. n. 412/’93) on the basis of heating degree days. Therefore, in these climatic zones, dwelling heating consumption represents the prevalent energy use, weighing significantly on the energy bills.

In 1997, the Municipality of Potenza was the first city of the Basilicata Region that adopted a Municipal Environmental Energy Plan (PEAC) in compliance with the national law 10/1991, which made the adoption of Local Energy Plan mandatory for cities with more than 50,000 inhabitants, aiming to characterize the municipal energy balances and plan energy-saving and renewable measures. 

With the adhesion to the Covenant of Mayors (occurred in February 2011) the Municipality of Potenza started its pathway towards an integrated approach to energy-climate policies, in the wake of the regional policy that started a process of transition towards a low-carbon and -greenhouse gas economy by integrating climate change mitigation and adaptation objectives in its programming (PIEAR - Environmental Energy Regulatory Plan, published on January 16th, 2010). As a result, the Sustainable Energy Action Plan of the Municipality of Potenza was released and approved on 6 February 2012.

The Municipality of Potenza is now in the process of adhering to the new Covenant of Mayors for Climate and Energy and will soon commit itself to achieving ambitious long-term objectives of mitigation and adaptation to climate change such as:

- The reduction in CO_2_ emissions (and possibly other greenhouse gases) on the territory of the signatory municipalities by at least 40% by 2030, by improving energy efficiency and greater use of renewable energy sources
- An increased resilience to the negative effects of climate change.

This political commitment will be translated into concrete actions outlined in a Sustainable Energy and Climate Action Plan (SECAP), which must be presented by the signatories, reporting every two years on the progress of their plans with respect to a Baseline Emission Inventory. This gives the Municipality of Potenza the opportunity to foster the adoption of quantitative tools to draw up an action plan for reducing energy consumption and CO_2_ emissions, starting from the municipal public buildings, which are very inefficient, and whose consumption represents a considerable share of the energy bill.

Taking into account this background, in the framework of the PrioritEE project, the main objectives of the Potenza case study were:

- Developing a methodology to help the municipal administration to elaborate a Local Energy Action Plan to improve energy efficiency (EE) and support renewable energy (RES) in municipal public buildings
- Characterizing the Municipal building stock, their energy consumptions and CO_2_ emissions by the application of the PrioritEE DSTool to prioritize EE and RES interventions
- Identifying feasible policy measures on short, medium and long term, drawing up a Local Action Plan
- Enabling a coordinated involvement of building users to facilitate and improve the implementation of strategic measures for the reduction in energy consumption in public buildings.

## 3. Results and Discussion

The first step of the procedure reported in Figure 2 (STEP A) concerned with identification of the “exemplar” municipal public buildings and their characterisation in terms of the DSTool parameters (e.g., energy consumption per energy uses, heating/cooling technologies, characterisation of the envelope). The Potenza Municipality runs a total of 62 municipal public buildings including 37 school buildings (among kindergartens, primary and middle schools) with a total annual natural gas consumption of 10.8 GWh (1,008,635 Smc), corresponding to a cost of 642,572 EUR (VAT included) and about 4428 t/year of CO_2_ emissions, an annual electricity consumption of 10.9 GWh, corresponding to a cost of 2,093,900 EUR (VAT included) and about 4469 t/year of CO_2_ emissions (reference year 2016). 

**Figure 3:**
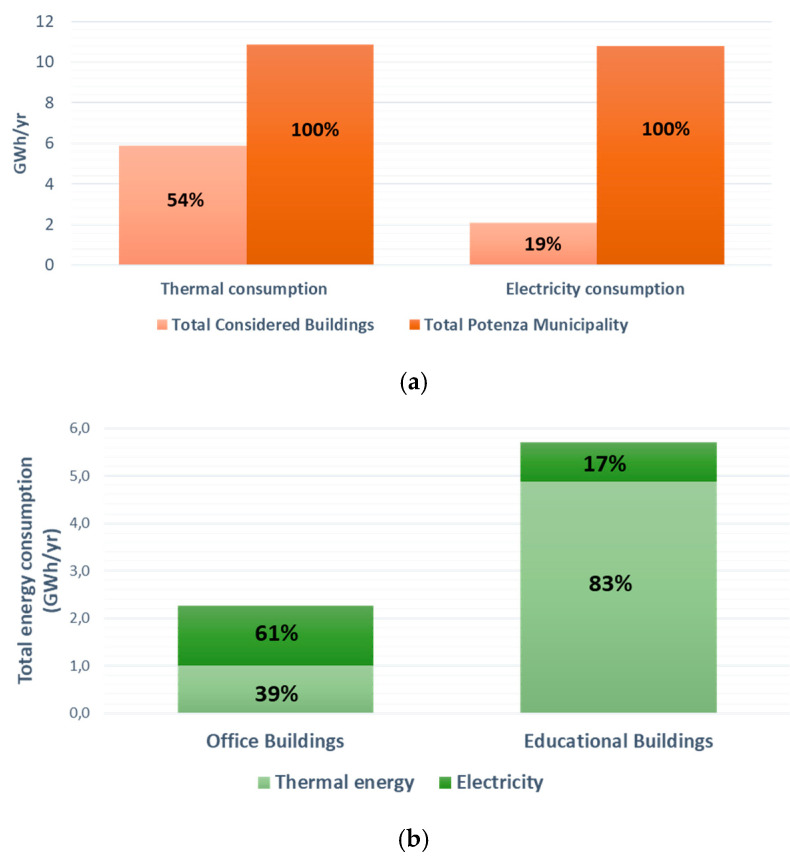
Thermal and Electricity Consumptions of the selected MPBs: (**a**) compared to total values for the Municipality of Potenza; (**b**) per typology.

The selection of the 25 exemplar buildings was carried out by a participatory process involving the municipal responsible and the technical staff. The sample buildings were chosen, taking into account their intended use (schools, office), the average number of occupants, the average consumption, and the age of buildings and energy class, where available, in order to cover a wide range of buildings and ensure representativeness. The DSTool was, therefore, tested on 21 schools and four office buildings (Table 1) with a total number of 5724 users (Table 1), representing 54% of the municipality’s natural gas and 19.3% of electricity consumption (Figure 3a). 

The energy consumption of the selected MPBs broken down by building typology is shown in Figure 3b, showing that, in Educational Buildings, the space heating consumption is higher (83%) while, in the offices, electricity consumption is prevailing (61%).

All the basic information on prevailing energy uses and infrastructure was gathered from municipal databases and archives (e.g., thermal and electricity consumption, heating system, utilised energy source for dwelling heating, lighting systems, presence of PV or other RES plants).

### 3.1. The Selection of EE and RES Interventions

The application of the Decision Support Tool (Step B, Figure 2) allows identifying a portfolio of EE and RES interventions for the selected MPBs providing a transparent and objective assessment of the various opportunities in terms of energy savings, reduction in CO_2_ emissions, investment costs and return on investment (ROI). 

The EE and RES interventions selected through the application of the DSTool are the following:

- Thermal insulation of external walls;
- Replacement of windows;
- Thermal insulation of roof;
- Installation of a ventilation system;
- Installation of a more efficient heating system;
- Installation of a more efficient cooling system;
- Installation of a more efficient lighting system;
- Installation of a solar hot water system;
- Installation of a photovoltaic (PV) system.

The potential energy saving for each building and for the individual energy efficiency measures intervention and types of RES plants are shown in Table 2. 

The results are given in terms of heat and electricity consumption, the most relevant indicators for benchmarking the different MPBs analysed. The DSTool representation is based on the chromatic logic of the traffic light: green cells indicate the most convenient measures in terms of energy saved, red cells highlight the least convenient options, whereas the yellow ones indicate the intermediate choice, that is, the technologies that could be reconsidered according to other indicators.

As shown in Table 2, the results obtained by analysing the selected buildings with the DSTool, show that insulation of the external walls, characterized by the green colour, represents the best energy efficiency intervention for all the considered buildings, followed by the installation of a PV system and improvements in the lighting system. The replacement of existing windows and the insulation of the roof, although less significant, can increase the energy savings achievable at a reasonable cost (Table 3), while the installation of a cooling system, a technology that is not currently used and is labelled as a “red option”, causes an increase in energy consumption.

As concerns the upgrading of the heating system, most of the selected buildings are already equipped with efficient plants integrating space heating and hot water heating systems (rated in the “A” energy efficiency category of the European energy efficiency label scheme [26]). Thus, it was assumed to replace the existing heating systems only for two buildings (the branches of the Busciolano Primary school located in the neighbouring villages of Giuliano and Avigliano Scalo) that are still equipped with old LPG plants (Table 2). 

**Figure 4:**
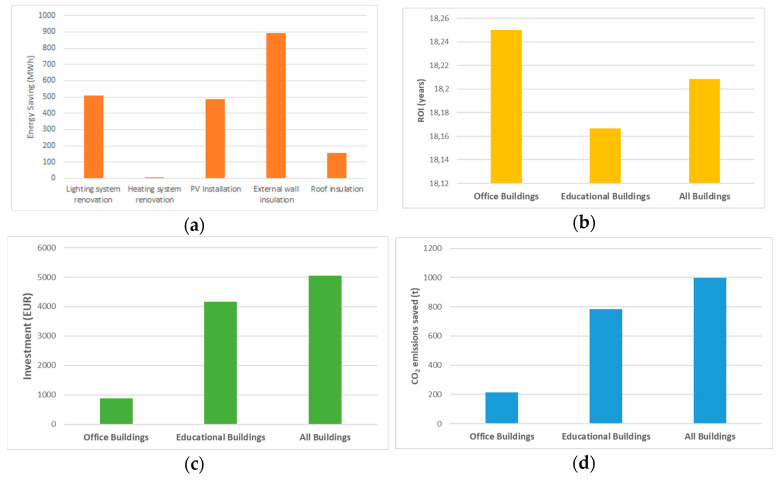
Effects of the combination of all the measures in terms of: (**a**) Energy Savings; (**b**) ROI, (**c**) investment cost, and (**d**) CO_2_ emission reduction.

Figure 4 summarises the results. The total energy savings achievable by implementing all the EE and RES options is about 4.07 GWh/year, corresponding to a total budget of about 5046 MEUR (Figure 4c) and a reduction in CO_2_ emissions of around 1 Kt/year (−30.65% of the CO_2_ emissions from MPBs considered) (Figure 4d) and a 0,49 GWh/year increase in RES-E generation. Energy Efficiency interventions on Educational Buildings allow savings of about 3211 kWh (Figure 4a), while the CO_2_ emissions reduction is about 785 t/year (Figure 4d). The Return on Investment (ROI) (Figure 4b) is around 18 years for both the building typologies (Figure 4b).

Taking into account the DSTool classification of the measures on the basis of the potential energy savings, the suitable EE and RES interventions by building typology were identified by considering the achievable CO_2_ emission reduction, the total investment costs, and the return on Investment (ROI), also provided by the analysis with the DSTool.

According to this analysis and following a cost-effectiveness criterium, the following interventions were identified as the most profitable:

- Renewal of the lighting system, PV installation in all buildings;
- Thermal insulation of the external walls in eight educational buildings;
- Roof insulation in seven educational buildings;
- Replacement of the heating system in two educational buildings.

**Figure 5:**
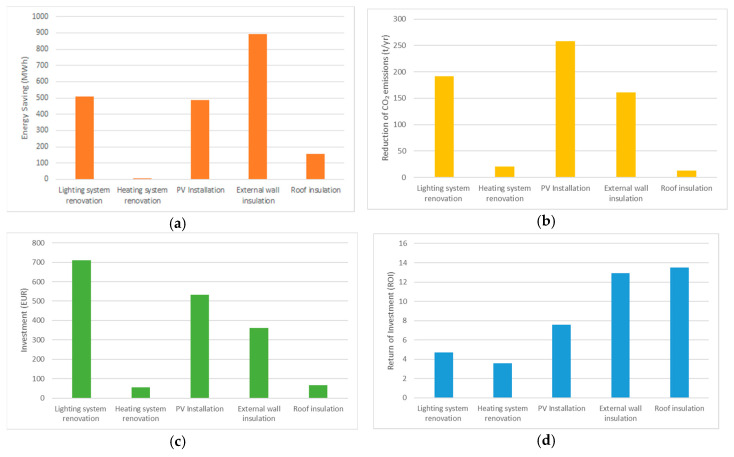
Total amount of (**a**) Energy Savings, (**b**) CO_2_ emission reduction, (**c**) ROI and (**d**) Investment cost per typology of intervention in the selected MPBs.

In total, the considered measures allow saving for about 2.05 GWh per year (heating and electricity consumption, Figure 5.) with an annual reduction in CO_2_ of around 6.44 t/year (−19.72% of the CO_2_ emissions from the tested MPBs), corresponding to a total cost of about 1,728,823 EUR.

### 3.2. The Local Action Plan of the Municipality of Potenza

The last step of the procedure is the definition of the Local Action Plan (Step E, Figure 2) based on the results achieved in previous steps. Considering the average annual investment of the Municipality of Potenza in energy saving measures (about 560,000 EUR, according to the 2019–2020 Municipal Forecast Budget), the required budget to implement all the measures will be available in about 12 years, therefore the implementation of all the proposed interventions is unfeasible in the short term.

Therefore, a three-year action plan was defined based on the current annual budget, for a total investment cost of 1,728,823 EUR, based on the following actions (Table 4):

1. ACTION #1: Renovation of the lighting system;
2. ACTION #2: Replacement of the heating system and installation of PV;
3. ACTION #3: Insulation of the external walls and roofs.

For the implementation of the Action Plan, a three-year period in the timeframe 2020–2022 was considered divided into three implementation phases: short, intermediate, medium term (Table 5). The implementation of the most efficient interventions in terms of energy savings and costs, considered high priorities for the Municipality, is foreseen in the short term phase.

The three blocks of actions are modular and can be implemented consecutively at a yearly pace in the three-year timeframe, according to the local authority necessities and budget availability, taking into account that the resources committed are in line with the annual expenses in the energy-saving sector of the Municipality of Potenza.

## 4. Conclusions

Climate crisis is the current challenge which countries and international organizations are called on to respond to urgently. The nexus between climate change and the built environment has been widely investigated, highlighting the consequences in terms of energy and other resources consumption and CO_2_ emissions. Therefore, there is an increasing necessity to define and implement mitigation and adaptation measures at national and local scales, with a key role of cities and small-scale communities. However, the definition and implementation of energy-climate policies is hampered by the lack of easy-to-use tools to support the decision-making process, and by the scarce concerns and motivation of stakeholders, including citizens.

In this framework, buildings represent a key sector to reduce energy consumption and CO_2_ emissions (e.g., [6,14]) and also to promote a participatory process that can contribute to fostering citizens’ awareness and enable new governance schemes.

The PrioritEE project contributed to these issues by developing a multitasking toolbox that provides a set of instruments addressed to the decision-makers and the community. Many tools, all freely available through the project website, were designed to support capacity buildings, increase knowledge and awareness on energy and climate issues, and assist public authorities in the drafting of a local action plan. Among them, the DSTool, an online calculator, can perform an evaluation of the effectiveness of energy renovation measures in public buildings ranking the measures by energy savings, CO_2_ emission reduction, investment costs and return on investment (ROI), either per each building or per group of buildings. Building typology, net heated area, primary source of energy, infrastructures and other technical parameters are considered to catalogue and analyse the building stock.

The application of the DSTool to the case study of the Municipality of Potenza presented here focused on 25 MPBs, involving a total number of about 5724 building users. Schools represented the majority of the investigated buildings, due either to their high consumption that has a relevant impact on the municipal energy bill or the possibility of enabling a participatory process to foster energy savings by behavioral changes [27]. A wide set of technical measures was considered, and the analysis performed with the DSTools put in evidence an energy saving potential 4.07 GWh/year, which can reduce CO_2_ emissions by 30.65% with respect to the actual level.

In line with other studies (e.g., [28]), the main retrofit measures included improvements in the building envelope (walls and roofs), heating system, lighting and the installation of PV. These measures were prioritized in a three-year implementation plan, taking into account the average annual expenditures in energy improvements in the Municipality of Potenza (around 560,000 EUR).

The municipal staff was involved in the selection of the buildings, in testing activities as well as in a short “hands-on” training on the use of the DSTool, promoting a participatory approach to governance and overcoming their initial mistrust due to the limited human resources dedicated to energy management issues and the time consuming process for data collection and analysis.

The DSTool has proven its effectiveness not only in the case study of the Municipality of Potenza, but also in all the other four applications to the case studies of the PrioritEE project. Its friendliness and immediacy in providing information on the classification of technologies in relation to different parameters constitutes its strength, making its use for personnel with different roles and levels of competence possible. Furthermore, the multiplicity of the parameters considered can make informed decisions on a diversified portfolio of options, evaluating their technical-economic effectiveness.

## Figures and Tables

**Table 1:** Main structural and energy characteristics of the 25 sample buildings selected for the Municipality of Potenza.

List of MPBs	Building Typology	Total Heated Area (m^2^)	Number of Floors	Year of Construction	Number of Users	Working Hours Per Year
1. City Hall	Office	974	4	1903	36	3432
2. Council Building	Office	335	3	1925	35	3432
3. Mobility Center	Office	6305	5	1988	171	1872
4. Municipal Offices	Office	3619	4	1975	184	1872
5. Leopardi-Kindergarten	School	720	1	1981	150	2100
6. Sinisgalli-Kindergarten	School	773	1	1984	150	2100
7. Sinisgalli-Middle School	School	2000	2	1984	378	2100
8. Busciolano-Primary School (Giuliano)	School	1514	1	1959	150	2100
9. Busciolano-Middle School (Via Sicilia)	School	1920	3	1969	491	2100
10. Busciolano-Kindergarten (San Nicola)	School	712	1	1993	500	2100
11. Busciolano-Primary School (Avigliano scalo)	School	336	2	1968	61	2100
12. Savio-Middle School	School	2700	3	1942	615	2100
13. Savio-Primary School	School	1496	2	1942	500	2100
14. Milani-kindergarten (Via Ionio)	School	1430	1	1983	150	2100
15. Milani-Kindergarten (Rossellino)	School	516	2	1968	49	2100
16. Milani-Middle School (Via Tirreno)	School	869	2	1977	255	2100
17. Milani- Primary and Middle School (Via Bramante)	School	1640	1	1994	255	2100
18. Torraca-Kindergarten	School	480	2	1980	74	2100
19. Torraca-Primary School (D. Viola)	School	356	3	1973	114	2100
20. Torraca-Primary School (18 Agosto Square)	School	1179	3	1888	255	2100
21. Leopardi-Primary School (Rodari)	School	1385	3	1981	255	2100
22. Leopardi-Primary and Middle schools	School	2700	3	1975	275	2100
23. La Vista-Primary School	School	856	1	1976	216	2100
24. La Vista-Middle School	School	2027	2	1976	255	2100
25. La Vista-Kindergarten	School	1089	1	1981	150	2100

**Table 2:** Energy savings achievable with the implementation of energy efficiency measures and renewable technologies for the selected Municipal Public Buildings (MPBs). The black background indicates measures that are not considered in the case study.

Buildings/IntervEntions	a. External Walls	b. Windows	c. Roof	d. VentilatIon System	e. Heating System	f. Cooling System	g. Lighting System	h. Solar Hot Water	i. PV
	Heat (kWh)					Electricity (kWh)			
City Hall	52,964	8946	13,565	1694	0	−5490	25,954	0	11,096
Council Building	22,636	3087	4599	687	0	−1890	8928	0	3192
Mobility Center	172,133	9243	44,145	5506	0	−35,466	72,906	0	167,757
Offices	131,560	37,435	33,744	4209	0	−20,363	41,851	0	41,268
Leopardi-Kindergarten	10,395	1822	1599	798	0	−1197	9686	0	5472
Sinisgalli-Kindergarten	57,005	10,014	8793	4376	0	−503	10,400	0	5890
Sinisgalli-Middle School	130,096	29,529	26,335	10,513	0	−4208	26,907	0	44,333
Busciolano-Primary School (Giuliano)	92,080	16,147	14,176	7069	14,966	−1153	20,369	0	11,514
Busciolano-Middle School (Via Sicilia)	90,606	21,126	18,341	10,460	0	−3131	25,831	0	18,240
Busciolano-Kindergarten (San Nicola)	50,877	8943	7805	4185	0	−1278	9579	0	5396
Busciolano-Primary School (Avigliano scalo)	18,177	4185	3682	1785	3500	−505	4520	0	3192
Savio-Middle School	269,943	61,272	54,644	21,815	0	−6745	36,324	0	25,650
Savio-Primary School	56,239	12,767	11,386	4545	0	−5792	20,126	0	14,212
Milani-kindergarten (Via Ionio)	101,642	17,812	15,637	7803	0	−997	19,238	0	10,868
Milani-Kindergarten (Rossellino)	65,871	15,365	13,340	7608	0	−698	6942	0	4902
Milani-Middle School (Via Tirreno)	37,310	8532	7540	3444	0	−971	11,691	0	8246
Milani-Primary and Middle School (Via Bramante)	53,434	9809	8221	6593	0	−829	22,064	0	12,464
Torraca-Kindergarten	14,796	3389	2995	1366	0	−2439	6458	0	4560
Torraca-Primary School (D. Viola)	10,427	2432	2112	1203	0	−1806	4783	0	3382
Torraca-Primary School (18 Agosto Square)	66,445	15,505	13,462	7671	0	−945	15,862	0	11,210
Leopardi-Primary School (Rodari)	75,396	17,099	15,248	6092	0	−1277	18,633	0	13,148
Leopardi-Primary and Middle schools	166,003	37,680	33,604	13,415	0	−1331	36,324	0	25,650
La Vista-Primary School	85,675	15,166	13,165	7517	0	−1075	11,516	0	6498
La Vista-Middle School	193,074	43,837	39,096	15,600	0	−2546	27,270	0	19,266
La Vista-Kindergarten	111,614	19,577	17,187	8569	0	−1368	14,651	0	8284

**Table 3:** Investment costs of energy efficiency measures and renewable technologies for the selected MPBs.

Buildings/Interventions	Investment Costs (EUR)								
	a. External Walls	b. Windows	c. Roof	d. Ventilation System	e. Heating System	f. Cooling System	g. Lighting System	h. Solar Hot Water	i. PV
City Hall	62,500	90,155	13,961	5000	8120	3260	18,270	0	12,167
Council Building	25,000	25,935	4016	5000	3724	1122	6285	0	3500
Mobility Center	212,500	449,350	80,410	18,750	42,033	21,058	118,219	0	183,944
Offices	125,000	257,925	34,616	18,750	30,161	12,090	67,862	0	45,250
Leopardi-Kindergarten	25,000	34,200	4590	5000	24,000	711	13,500	0	6000
Sinisgalli-Kindergarten	25,000	36,813	4941	5000	25,767	299	14,494	0	6458
Sinisgalli-Middle School	75,000	118,750	21,250	8750	33,333	2499	37,500	0	48,611
Busciolano-Primary School (Giuliano)	62,500	71,963	9659	6250	50,467	684	28,388	0	12,625
Busciolano-Middle School (Via Sicilia)	62,500	114,000	15,300	7500	21,333	1859	36,000	0	20,000
Busciolano-Kindergarten (San Nicola)	25,000	33,725	4526	5000	23,733	759	13,350	0	5917
Busciolano-Primary School (Avigliano scalo)	12,500	19,950	2678	5000	5600	300	6300	0	3500
Savio-Middle School	100,000	160,313	21,516	21,250	30,000	4005	50,625	0	28,125
Savio-Primary School	50,000	88,825	11,921	5000	24,933	3439	28,050	0	15,583
Milani-kindergarten (Via Ionio)	50,000	67,925	9116	7500	47,667	592	26,813	0	11,917
Milani-Kindergarten (Rossellino)	12,500	30,638	4113	8750	8600	415	9675	0	5375
Milani-Middle School (Via Tirreno)	25,000	51,538	6918	5000	14,483	577	16,294	0	9042
Milani-Primary and Middle School (Via Bramante)	62,500	77,900	10,455	5000	54,667	492	30,750	0	13,667
Torraca-Kindergarten	12,500	28,500	3825	5000	8000	1448	9000	0	5000
Torraca-Primary School (D. Viola)	12,500	21,138	2838	5000	3950	1073	6666	0	3708
Torraca-Primary School (18 Agosto Square)	37,500	70,063	9404	6250	13,100	561	22,106	0	12,292
Leopardi-Primary School (Rodari)	50,000	82,175	11,029	6250	15,389	758	25,969	0	14,417
Leopardi-Primary and Middle schools	100,000	160,313	21,516	18,750	30,000	790	50,625	0	28,125
La Vista-Primary School	37,500	40,613	5451	7500	28,533	638	16,050	0	7125
La Vista-Middle School	75,000	120,413	16,161	18,750	33,783	1512	38,006	0	21,125
La Vista-Kindergarten	37,500	51,775	6949	8750	36,300	812	20,419	0	9083

**Table 4:** Features of the selected actions. The percentages of CO_2_ emission reduction are calculated compared to the total CO_2_ emission from 25 selected buildings of the Municipality of Potenza (3262.9 t/year).

Actions	Savings (kWh)	Reduction of CO_2_ Emissions (t/year)	Investment (EUR)
#1	508,812	191.31 (5.86%)	711,214
#2	492,570	278.45 (8.53%)	588,622
#3	1,048,730	173.80 (5.33%)	429,026
Total	2,050,112	643.56 (19.72%)	1,728,863

**Table 5:** Timeframe for the implementation of the Actions. The “X” indicates the type of actions implemented in each time period.

Actions	Implementation Timeframe		
	Short (2020)	Intermediate (2021)	Medium (2022)
#1	X		
#2		X	
#3			X
